# Study protocol of a pilot randomized controlled trial of transcranial direct current stimulation paired with reappraisal training for treatment of cannabis use disorder

**DOI:** 10.1371/journal.pone.0328205

**Published:** 2025-08-28

**Authors:** Adam M. Stryjewski, Tammy J. Chung, Li Yan McCurdy, Tara L. Spitzen, Kangxin Zhang, Kristen P. Morie, Vaughn R. Steele, Michael J. Crowley, Marc N. Potenza

**Affiliations:** 1 Department of Psychiatry, Yale School of Medicine, New Haven, Connecticut, United States of America; 2 Department of Radiology and Biomedical Imaging, Yale School of Medicine, New Haven, Connecticut, United States of America; 3 Institute of Living, Hartford Hospital/HealthCare, Olin Neuropsychiatry Research Center, Hartford, Connecticut, United States of America; 4 Center for Brain & Mind Health, Yale University, New Haven, Connecticut, United States of America; 5 Child Study Center, Yale School of Medicine, New Haven, Connecticut, United States of America; 6 Department of Neuroscience, Yale School of Medicine, New Haven, Connecticut, United States of America; 7 Wu Tsai Institute, Yale University, New Haven, Connecticut, United States of America; 8 Connecticut Mental Health Center, New Haven, Connecticut, United States of America; 9 Connecticut Council on Problem Gambling, Wethersfield, Connecticut, United States of America; PLOS: Public Library of Science, UNITED KINGDOM OF GREAT BRITAIN AND NORTHERN IRELAND

## Abstract

**Introduction:**

As legalization of cannabis products continues, cannabis use becomes more prevalent, and concerns regarding cannabis use disorder (CUD) rise, improving CUD treatment has become increasingly important. Techniques used to regulate emotions, such as cognitive reappraisal, may help manage cravings for cannabis in individuals with CUD. Transcranial direct current stimulation (tDCS), a noninvasive brain stimulation technique, may improve regulation of emotions and reduce substance use. This study aims to determine whether the addition of tDCS to training in cognitive reappraisal leads to greater reductions in cravings and cannabis use than cognitive reappraisal without active stimulation.

**Methods and analysis:**

This longitudinal between-subjects study will recruit 60 participants who will each be randomly assigned to receive either active or sham tDCS. Participants will undergo 5 sessions, each spaced approximately one week apart. In session, they will receive 20 minutes of (active/sham) 1.5mA anodal stimulation over the right dorsolateral prefrontal cortex while receiving training in cognitive reappraisal. Primary outcomes include changes in cannabis use during the study, changes in electroencephalogram brain activity when viewing cannabis cues, and changes in cannabis craving intensity.

**Discussion:**

The results of this study will inform a full-scale randomized controlled trial designed to assess the effectiveness of this intervention. More broadly, these results will add to the literature on the role of tDCS in enhancing CUD treatment.

**Trial registration:**

ClinicalTrials.gov NCT06369311

## Introduction

As the legalization of cannabis products continues with cannabis use becoming more prevalent, concerns regarding cannabis use disorder (CUD) have been increasing [1]. Concurrently, efforts continue towards improved CUD treatment [2–6]. Craving, a strong desire to use a substance or engage in a behavior, is a defining feature of substance use disorders, including CUD [7]. Craving has been linked to treatment outcomes [8,9], where stronger cravings are typically associated with poorer treatment outcomes [2,10,11].

Since craving may be considered a motivational state that can be regulated [12,13], identifying ways to reduce craving is a common target of evidence-based treatments such as cognitive behavioral therapy and mindfulness-based approaches [3]. One way of regulating craving involves cognitive strategies such as cognitive reappraisal. In the context of CUD, cognitive reappraisal can entail thinking about the negative consequences of engaging in behaviors like using substances [9,12,14–16]. Use of reappraisal strategies in the laboratory has been associated with reduced craving and negative affect among multiple clinical groups, including those who use tobacco [17], cocaine [18], and opioids [4]. Reappraisal training has also been found to be effective for reducing craving for cannabis [5,19] and reducing cannabis use [19–21], making it a promising strategy for enhancing regulation skills, minimizing cravings, and improving treatment outcomes. However, effect sizes tend to be modest. Thus, approaches to enhance reappraisal effects warrant further investigation.

Transcranial direct current stimulation (tDCS), a noninvasive application/administration of electrical current to modulate brain activity and potentially enhance neuroplasticity [22,23], may enhance cognitive reappraisal and craving regulation when applied to the dorsolateral prefrontal cortex (dlPFC). Indeed, the dlPFC has been implicated in regulation of craving [14,24–26]. Preliminary data from clinical research indicates that dlPFC tDCS may be effective in reducing substance use for individuals with alcohol use disorder [27–29], tobacco use disorder [30,31], and CUD, although findings for CUD have been limited by a small sample size (8–9 participants per condition) [6]. Specifically, the CUD study found that participants who received a single session of tDCS to the dlPFC had greater reductions in cannabis craving compared to those who received sham stimulation [6]. Therefore, it is possible that these reductions in craving may be amplified when combining dlPFC-targeted tDCS and training in cognitive reappraisal for CUD.

In addition to its potential to enhance craving regulation, tDCS offers a unique opportunity when paired with electroencephalography (EEG) to explore underlying neurophysiological mechanisms. EEG is a noninvasive, high-temporal-resolution method for measuring brain activity, well-suited for tracking dynamic processes involved in craving and cognitive regulation. Specifically, event-related potentials (ERPs) such as the P300 and late positive potential (LPP) provide sensitive indices of attentional and affective responses to substance-related cues, with increased amplitudes reflecting greater salience or emotional engagement [32–34]. Moreover, frontal theta power, an oscillatory feature associated with cognitive control, has been implicated in the neural implementation of strategies to regulate craving [35]. Integrating EEG into this study may allow for objective assessment of changes in neural features linked to exposure to cannabis-related cues, offering insight into how cognitive reappraisal and tDCS may interact to modulate brain function. Together, the use of EEG and tDCS provides a multimodal framework for both enhancing and monitoring the regulation of cannabis craving in individuals with CUD.

The present study seeks to evaluate the efficacy of tDCS to the right dlPFC paired with training in cognitive reappraisal in improving craving regulation and reducing cannabis use in individuals with CUD. Specifically, we hypothesize that participants who receive active versus sham tDCS will have 1) greater reductions in daily cannabis use, 2) changes in neural correlates of EEG features associated with craving (e.g., relatively diminished LPP amplitude and increased delta power) while viewing cannabis cues, and 3) greater reductions in cannabis craving. We also hypothesize tDCS-related increases in neural correlates of EEG features associated with regulation (e.g., frontal theta power).

## Materials and methods

### Study design

This protocol is written in accordance with the Standard Protocol Items: Recommendations for Interventional Trials (SPIRIT) reporting template [36]. The present study will utilize a randomized design where participants are allocated to either an active stimulation condition or a sham control condition in a 1:1 ratio. To ensure randomization of active/sham administration across sexes, this 1:1 ratio is allocated within males and females separately. To minimize bias, both study participants and research staff running study visits will be blinded to the participant’s assigned experimental condition. This entails five weekly visits (baseline and four weekly visits thereafter), during which participants will undergo a 20-minute session of (active/sham) tDCS and reappraisal training each week. Participants will complete a self-report measure of cannabis craving [37] immediately before and after each tDCS and reappraisal training session. Before the first tDCS session on the first visit and after the last tDCS session on the fifth visit, participants will complete the Regulation of Craving (ROC) task [3,38], during which EEG activity will be recorded. In addition to the in-person study visits, beginning after the first study visit and ending at the last visit, participants will provide information on past 24-hour cannabis use through a daily survey delivered via short message services (SMS).

### Participants

#### Eligibility criteria.

Participants will consist of 60 adults between the ages of 18 and 50 years who reside in the Greater New Haven area in Connecticut, USA and meet criteria for moderate or severe CUD. Diagnostic criteria for CUD will be assessed during screening procedures utilizing a symptom checklist based on the Diagnostic and Statistical Manual for Mental Disorders, 5^th^ Edition (DSM-5) criteria for CUD. Participants must meet at least four of these criteria to be eligible for the study. Additionally, participants must be looking to reduce their cannabis use and must have a positive urine toxicology screen for metabolites indicating regular cannabis use. Participants must also have the capacity to provide informed consent for the study.

Participants may be excluded from the study for several reasons. First, participants may not be receiving any other form of treatment for CUD while they are participating. Lifetime history of serious neurological (e.g., dementia, multiple sclerosis, tumors) or psychiatric (e.g., psychosis, bipolar disorder) conditions, strokes, or losses of consciousness will preclude participants from being eligible. Participants will also be excluded if they have a current medical history of non-CUD substance use disorders. Several contraindications to tDCS will also prevent participants from safely participating, including the presence of metallic implants or electronic devices (e.g., heart pacemakers), any history of seizures, and pregnancy or a plan to become pregnant during the duration of the study. Finally, participants will be excluded for endorsing suicidal or homicidal thoughts, using nicotine equivalent to one pack of cigarettes per day or more, or using exclusionary substances, including amphetamines, benzodiazepines, methamphetamine, cocaine, and opioids. Participants who primarily use cannabis in the form of edibles will be excluded, as the ROC task for CUD utilizes cannabis images related to smoking and vaping and does not include pictures of edibles.

At the start of each visit, the study team will collect biological verification of tetrahydrocannabinol (THC) through urine and salivary toxicology screening. Urine toxicology screening will be completed at each visit to ensure participants meet the full inclusion criteria for the study, in that participants need to both test positive for the presence of THC and negative for exclusionary substances (e.g., opioids, cocaine, methamphetamine). Salivary toxicology screening will be completed at the start of each study visit to ensure that participants abstain from cannabis use prior to each study appointment (i.e., as demonstrated by THC concentration of ≤10 ng/ml). Participants with a positive salivary toxicology screen will be asked to reschedule their visit for another day.

### Recruitment and informed consent

Participants will primarily self-refer to this study through flyers posted in the community, clinical trial listings regularly emailed to a pool of prospective participants through the Yale Center for Clinical Investigation (YCCI), and trial listings on internet resources (e.g., ClinicalTrials.gov). Prospective participants will be screened by a research assistant to determine eligibility. If a participant is eligible, study staff will schedule their first study visit. Participants will receive an electronic copy of the consent form to review prior to their first appointment. Prior to any research data being collected, a research assistant will discuss the study in detail with prospective participants and assess each participant’s capacity to provide informed consent using a brief questionnaire on important details of the study. Once capacity to provide consent is determined, the participant and research team member will sign the consent form, and the participant will be provided a copy for their records. The consent form and questionnaire assessing capacity to provide consent are included as supporting materials. Recruitment has not yet started and is anticipated to take place from April 2025 to April 2026.

### Participant timeline

**Fig 1:**
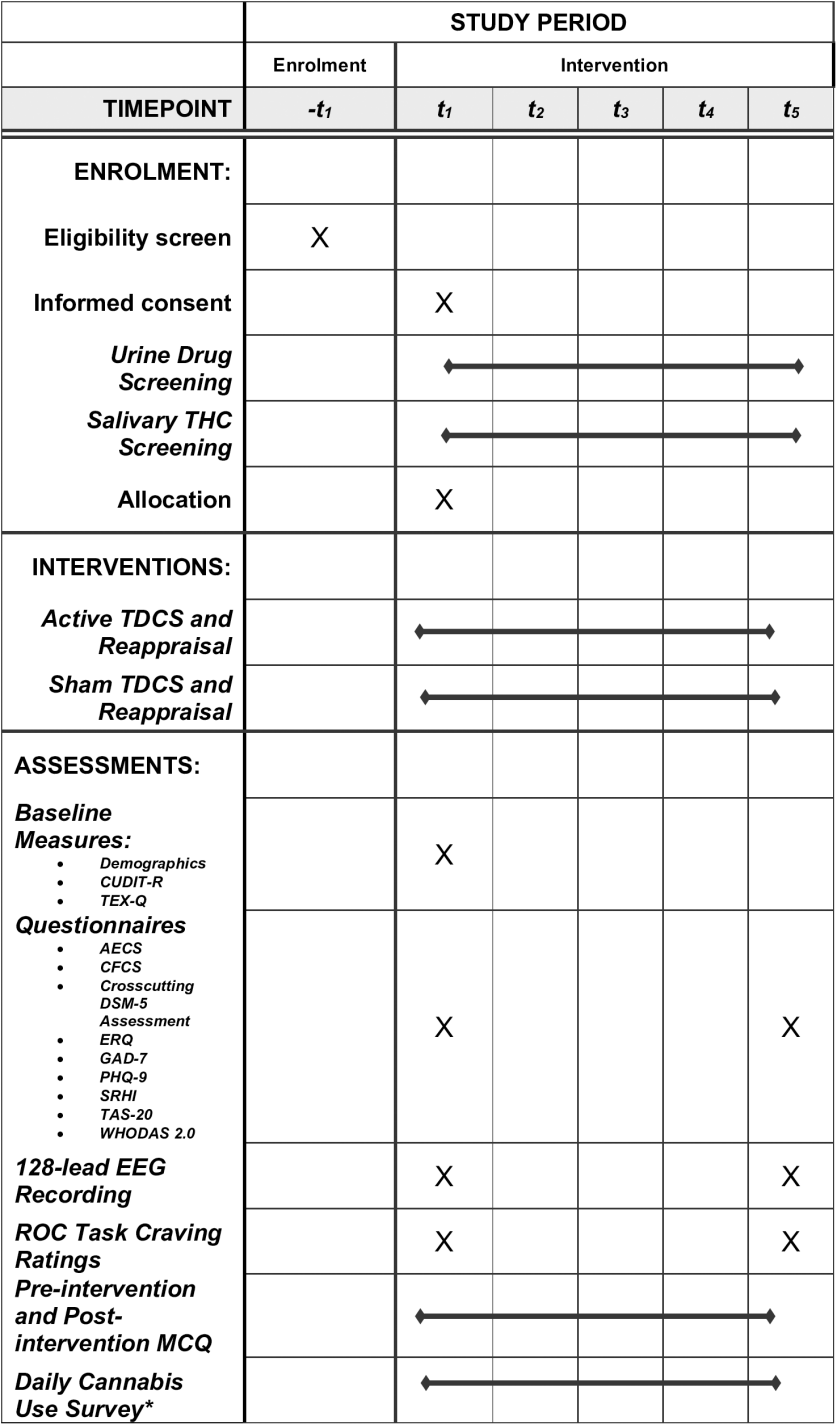
Notes: AECS: Anticipated Effects of Cannabis Scale; CFCS: Consideration for Future Consequences Scale; CUDIT-R: Cannabis Use Disorder Identification Test, Revised Edition; ERQ: Emotion Regulation Questionnaire; GAD-7: Generalized Anxiety Scale; PHQ-9: Patient Health Questionnaire; MCQ: Marijuana Craving Questionnaire; SRHI: Self-Report Habit Index; ROC task: Regulation of craving task. TDCS: 20 minutes of 1.5mA active/shame transcranial direct current stimulation; TAS-20: Toronto Alexithymia Scale-20; TEX-Q: Treatment Expectations Questionnaire; WHODAS 2.0: World Health Organization Disability Assessment Schedule 2. Note that questionnaire completion and the ROC task with EEG recording occur before the intervention session on V1, and after the intervention session on V5. * Daily cannabis use surveys are sent to each participant for 28 days following enrolment.

The timeline for participants is presented in Fig 1. After participant eligibility is determined, participants will be invited to the first study visit (V1), where they will provide informed consent for participation, complete baseline questionnaires, complete a pre-intervention ROC task while EEG activity is recorded, and then receive the first session of tDCS and reappraisal training. Participants will also complete the Marijuana Craving Questionnaire (MCQ) [37] before and after the intervention. This visit should take about three hours.

Study visits two through four (V2-V4) will be shorter visits during which participants only receive tDCS and reappraisal training and complete the MCQ before and after the intervention. Study visit five (V5) will consist of the same intervention administration and assessment of cannabis cravings as all previous visits. Additionally, participants will complete a post-intervention ROC task while EEG activity is recorded followed by post-intervention questionnaires.

At every appointment, research staff will assess for any adverse events (AEs) and record those reported by each participant in their study record. The study investigators will be responsible for determining the severity of AEs and their relationship to study procedures. Following any severe AEs that are possibly, probably, or definitely related to study procedures, research staff will consult the investigative team to determine if it is appropriate to continue study procedures. Although we do not expect any serious adverse events (SAEs), any participant who reports an SAE will be withdrawn from the trial and referred for appropriate follow-up care. Additionally, following the first study visit and until the end of the last study visit, participants will receive a once-daily survey asking about the amount of cannabis used and the form (e.g., smoked, vaped, edibles) of cannabis used in the past 24-hour window. In this survey, participants will also rate how the amount of cannabis used compares to the amount of cannabis they would typically use.

### Participant compensation

Participants will receive up to $300 in compensation for completing the study. Specifically, participants will receive $50 for completing V1, $25 for completing each of V2-V4, and $50 for completing V5, with a $75 bonus for completing all five study visits. Participants will also receive $1 for each daily cannabis use survey that they complete (up to $28) with a $3 bonus for each week in which surveys are completed on all 7 days (up to $12). Finally, participants can earn a $10 bonus for completing 75% (i.e., at least 21 out of 28) of the daily surveys. All compensation will be paid to the participants in cash on the day of each study visit.

### Intervention

All tDCS interventions will be delivered using the Soterix Medical 1x1 tDCS Clinical Trial system. Upon study enrollment, each participant will be randomly assigned to an active or sham stimulation group. In the active group, each participant will receive a 1.5mA stimulation over 20 minutes with a 30-second ramp-up and ramp-down time. In the sham control group, each participant will not receive any active stimulation over the 20-minute intervention apart from the ramp-up and ramp-down time. The ramp-up and ramp-down period in the sham stimulation is designed to make participants feel the same itching sensation as participants in the active stimulation group. Direct current stimulation will be delivered through a pair of carbonated silicone electrodes enclosed in an absorbent pad containing 10mL of 0.9% NaCl saline solution. For anodal stimulation of the right dlPFC, the anode electrode will be placed on F4 in accordance with the 10–20 international EEG system [39] and the cathode will be placed on the left upper deltoid. The Soterix Medical 1x1 Clinical Trial device comes equipped with site-specific stimulation codes that will be known only to one individual on the investigative team (VRS), such that all others will be blind to the assigned condition for the study duration. In the event that participants experience an AE related to study procedures, participants may be unblinded in order to facilitate follow-up care.

While tDCS is being administered, all participants will receive reappraisal training to learn to regulate their cravings. Each tDCS session entails approximately 10 minutes of psychoeducation on concepts and strategies that can help them regulate their cravings (e.g., reappraisal), followed by 10 minutes of practicing evoking negative consequences of cannabis use while viewing cannabis images. For example, during the first session, participants will be asked to briefly share their reasons for wanting to reduce their cannabis use with the research assistant, who will then outline several well-documented, long-term adverse effects of cannabis use, spanning physical, psychological, and occupational domains. Next, participants will generate a list of 3–5 specific and personally relevant negative consequences of cannabis use, which the research assistant will write down. The research team will utilize this information during each weekly visit to work with the participant during the reappraisal training. Participants are then introduced, in lay terms, to the idea of cognitive reappraisal with examples of how they may already be using this strategy in everyday life (e.g., consequences of not paying bills, sending angry texts). Next, participants are shown how they can use this strategy when they see things that remind them of cannabis (introduced as triggers) or when they have urges to use cannabis. They are given the rationale that, with practice, thinking about the negative consequences of using can help reduce cravings to use cannabis and reduce cannabis use. After the psychoeducation portion, participants will spend approximately ten minutes practicing this skill by viewing 20 images of cannabis and following instructions to passively view half of the images and verbalize their negative consequences for the other half of the images. Research assistants may ask participants to elaborate with prompts such as, “How would that make you feel if that [negative consequence] occurred?” Participants can refer to their list of negative consequences during this practice. Participants will rate how much they crave cannabis after viewing each image on a 5-point scale, similar to the ROC task (described in Primary Outcomes below).

### Measures

#### Baseline measures.

Participants will complete the following measures as part of their baseline assessment on V1. The Demographics form asks participants to provide information on their age, sex, race, ethnicity, and handedness. The Cannabis Use Disorder Identification Test, Revised Edition (CUDIT-R) is an 8-item questionnaire designed to identify cannabis misuse in the past 6 months [40]. The revised version has demonstrated comparable psychometric properties to the original scale and high rates of sensitivity and specificity in identifying cannabis misuse [40]. The Treatment Expectation Questionnaire (TEX-Q) is a 15-item questionnaire that measures participants’ expectations of medical and psychological treatments [41].

### Questionnaires

Participants will complete the following questionnaires at both their first and last study visits: The Anticipated Effects of Cannabis Scale (AECS) is a 17-item measure that assesses both positive and negative effects of cannabis that participants may expect [42]. The AECS further considers these expectancies based on the valence (positive versus negative) and arousal (high versus low) characteristics of each possible expectation [42]. The Consideration for Future Consequences Scale (CFCS) is a 12-item questionnaire that assesses the extent participants value immediate rewards over long-term consequences and has demonstrated acceptable levels of validity and reliability [43]. The DSM-5 crosscutting assessment assesses mental health domains that are relevant to psychiatric diagnoses [44]. The Emotion Regulation Questionnaire (ERQ) is a 10-item scale that measures participants’ tendencies to regulate their emotions via cognitive reappraisal and expressive suppression [45]. The Generalized Anxiety Disorder (GAD-7) is a 7-item clinical measure for assessing GAD [46]. The Patient Health Questionnaire (PHQ-9) is a measure of depression severity [47]. The Self-Report Habit Index (SRHI) is a 12-item questionnaire which assesses common characteristics of habitual behaviors [48]. The SRHI shows good evidence for reliability and validity across a range of substance use disorders, including CUD [48]. The Toronto Alexithymia Scale (TAS-20) is a 20-item questionnaire that evaluates an individual’s ability to identify and distinguish emotions [49]. The revised 20-item measure has demonstrated a three-factor structure and good internal consistency [49]. Finally, the World Health Organization Disability Assessment Schedule 2 (WHODAS 2.0) measures functioning and disability [50].

### Primary outcomes

The first primary outcome is cannabis use during the study. This will be measured through a brief daily survey. Participants will be sent a link to a REDCap survey at 8:00am daily to report on cannabis use over the last 24 hours. Participants will be asked about the amount of cannabis used, the form of cannabis used (e.g., smoked, vaped), and how the amount used compares to their typical use amount on a 5-point Likert scale, where 1 indicates much less cannabis used than usual, 3 indicates the usual amount of cannabis was used, and 5 indicates much more cannabis used than usual.

**Fig 2:**
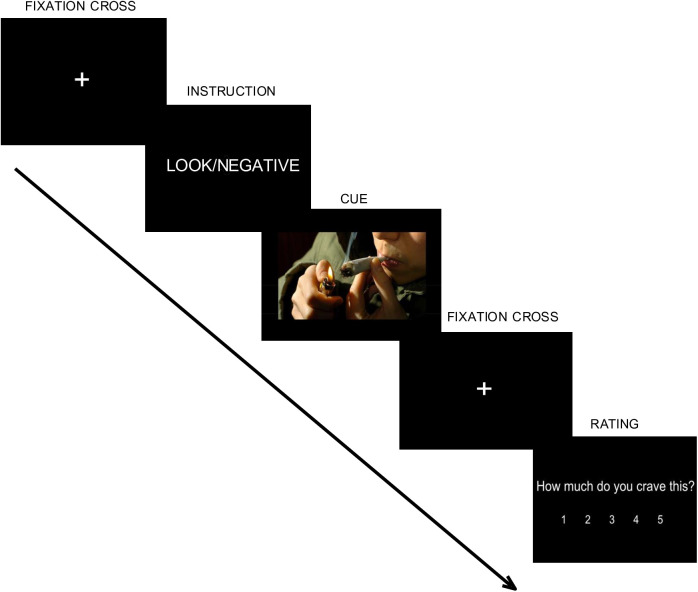
Schematic representation of a single trial of the ROC task. Participants will view a fixation cross for 3 seconds before the instruction (LOOK/NEGATIVE) for participants is displayed for 3 seconds. A cannabis image will be displayed for 6 seconds, during which participants are to either look at the image (“LOOK”) or think about the negative consequences of using cannabis (“NEGATIVE”). After a 3-second fixation cross, participants will have up to 10 seconds to rate their level of craving for cannabis on a 5-point scale. Participants will complete 50 trials, comprising 25 LOOK trials and 25 NEGATIVE trials.

The second primary outcome is the EEG correlates of craving and regulation during the ROC task (Fig 2), which participants will complete before the first intervention session and after the fifth intervention session. The ROC task utilizes E-Prime software to present participants with a series of validated cannabis images, including pictures of the cannabis plant and images depicting various cannabis primary use methods [51]. The ROC task consists of 25 LOOK and 25 NEGATIVE trials total, presented in alternating LOOK and NEGATIVE blocks of 5 trials each (i.e., 5 LOOK trials, followed by 5 NEGATIVE trials, followed by 5 LOOK trials, etc.). During the LOOK trials, participants will be instructed to simply look at the image; during the NEGATIVE trials, participants will be instructed to think about the negative consequences of using cannabis. EEG data will be collected using a 128-lead Electrical Geodesic EEG system, with gel-based collection, sampled at 1000 Hz.

Our rationale for ERP component selection and labeling is grounded in the literature on emotional image processing. In picture-viewing paradigms involving affectively salient stimuli, the early neural response often overlaps with the P300 in both timing and topography. However, unlike discrete stimuli used in tasks like oddball tasks, emotional images typically evoke more complex and variable cognitive appraisal processes, leading to temporal jitter in the onset of peak neural engagement across trials. This results in a less sharply defined P300 and the emergence of a sustained slow-wave positivity that blends with the LPP over centro-parietal regions. To align with conventions in the ERP literature, we assess the P300 in the ~ 250–300 ms window and the LPP in the 600–900 ms window. Thus, mean amplitudes of the P300 (~250–300 ms following cannabis cue) and the early LPP (600–900 ms following cannabis cue) will be quantified. Additional analysis will also assess and consider frontal theta power.

The third primary outcome is self-reported levels of cannabis craving. This will be assessed using the MCQ [37], which will be administered immediately before and after each tDCS and reappraisal training session at each study visit. It will also be assessed using the ROC task on V1 and V5: self-reported levels of craving in response to cannabis images will be averaged separately across all LOOK trials and all NEGATIVE trials and compared.

### Data collection, management, and monitoring

#### Data collection.

Self-report data will be collected primarily through standardized assessments administered to participants through Yale University’s 21 CFR part 11 compliant version of REDCap. Participants will complete these measures during each study visit and all data collected will be identified by an ID number unique to each participant. EEG data will be collected through a 128-lead Geodesic EEG net, as described above.

### Data management

The principal investigators (MJC, MNP, VRS) will oversee data management. Only study team members who have been trained in human subject research and Good Clinical Practice will have access to the data. All data will be stored on devices that are compliant with the Health Insurance Portability and Accountability Act (HIPAA) and 21 CFR part 11 guidelines for electronic data collection and approved by the Yale Human Investigation Committee (HIC). Deidentified research data will be stored on Yale-managed study computers. Information containing identifiers (e.g., consent forms, participant contact information, master list) will be stored securely in locked filing cabinets at the Yale School of Medicine and will only be accessible to authorized research staff.

### Data monitoring

The principal investigators are responsible for monitoring data, ensuring compliance, and conducting safety reviews throughout the course of the study. Recruitment, retention rates, and availability of primary outcome data will be discussed at least once monthly.

### Data analysis

Data collected throughout this study will primarily be analyzed using SPSS software. To address the first aim of the study, reductions in cannabis use, as reported by research participants through the daily-administered REDCap surveys, will be analyzed using a multilevel modeling strategy. Multilevel modeling with maximum likelihood estimation for missing data will be used to conduct a linear analysis of daily use of cannabis using a nested hierarchical structure with both between- and within-subject predictors. The main effects of Group (i.e., stimulation versus sham) and Time, as well as the Group x Time interaction will be the primary fixed effects of interest. Daily use of cannabis will be compared across the study weeks, and multilevel models will be constructed to examine the slopes of longitudinal data across the weeks.

Statistical analysis of the EEG correlates of craving and regulation will be conducted using Netstation pipelines for ERPs and Matlab scripts for oscillatory power. Data will be filtered offline at 0.1–30 Hz. Ocular artifact correction will be performed on the mean amplitudes of each component with a specified timeframe. The components of interest for this analysis will consist of the P300, the LPP, and frontal theta power. These variables will be compared across Group (i.e., stimulation versus sham), Condition (i.e., LOOK or NEGATIVE), and Time (V1 and V5) in a series of ANOVAs. We hypothesize that active stimulation will result in heightened amplitude of the early LPP in the LATER condition, as regulation of craving is hypothesized to be enhanced, and diminished amplitude of the late LPP in the LATER condition, as arousal related to craving is hypothesized to be reduced. Further, we expect that frontal theta power will be enhanced after tDCS stimulation during the LATER condition.

For the third primary outcome, craving will be measured both pre- and post-tDCS using the MCQ [37], and active and sham groups will be compared via an ANOVA (Group x Condition x Time). Additionally, self-reported cravings elicited by cannabis images in the ROC task in both the LOOK and NEGATIVE conditions are rated on a 5-point Likert scale at baseline and post-treatment. These data will be averaged across trials and compared between groups via an ANOVA (Group x Condition x Time).

### Study status

This study will involve human participants and approval for this study was obtained from the Yale University Human Subjects Committee (HIC#2000037345). A Certificate of Confidentiality was awarded through the National Institutes of Health. The trial is registered on ClinicalTrials.gov (NCT06369311). Recruitment and data collection have not begun, and recruitment is expected to begin in April 2025. Data collection, analysis, and study completion is expected in April 2026.

## Discussion

This study will extend the available literature by determining the efficacy of tDCS stimulation in reducing cannabis craving when paired with reappraisal training. A multi-method approach utilizing quantitative self-report data and brain measures of craving and regulation will allow the research team to fully investigate the efficacy of the intervention. All major amendments to the protocol will be submitted to the National Institute of Drug Abuse (NIDA) for preauthorization and will then be documented and submitted to the Yale University HIC for approval. Following approval, these changes will be reflected in the trial registry in ClinicalTrials.gov. Research findings will be disseminated through peer-reviewed journals and conference presentations. Further, data collected in this study will become available to the academic community through the National Institute of Mental Health Data Archive (NDA) following study completion.

Results of this study will contribute to the scientific literature on tDCS and its clinical uses. Additionally, there are clinical implications in that techniques that result in large reductions in cannabis cravings may provide low-risk intervention for individuals struggling with CUD. Given that cannabis legalization has become increasingly common across the United States and Europe, developing more effective treatments for CUD is important, as increased access to cannabis may lead to an increase in incidence of CUD [1].

The present study has some expected limitations. First, as research participants are being recruited primarily in the Greater New Haven area, findings may not generalize to other regions. Second, our study will exclude participants who primarily use cannabis in the form of edibles, as the cannabis cue stimulation set used in our study does not include images of edibles. For individuals who primarily use cannabis via edibles, pictures of the cannabis plant or other forms may not elicit the same emotional and physiological responses as they would for those that use cannabis via smoking or vaping. Although excluding participants who exclusively use edibles may limit the generalizability of the findings, this approach limits heterogeneity and facilitates study of the effects of cannabis cues in most individuals using cannabis, since smoking and vaping are more common methods of cannabis use than is consuming edibles [52].

Despite limitations, the present study serves as an important step for investigating the potential benefits of utilizing tDCS in the treatment of CUD, which can guide future studies in tDCS that focus on clinical populations who are seeking treatment for CUD. Results from this study may provide evidence for an innovative neurostimulation technique as a potential augmentation strategy for treatment of CUD and will provide preliminary data for future work investigating tDCS in CUD treatment settings.
