# Supplementary material for: Study protocol of a pilot randomized controlled trial of transcranial direct current stimulation paired with reappraisal training for treatment of cannabis use disorder
Source: PLoS One. 2025 Aug 28;20(8):e0328205. doi: 10.1371/journal.pone.0328205 (PMC12393744; doi:10.1371/journal.pone.0328205)
Supplement: Attachment — Submitted filename: Potenza PLoS One letter 050525.docx [file pone.0328205.s001.docx]

**MARC N. POTENZA, M.D., Ph.D.** *Director, Division on Addictions at Yale*

*Director, Yale Center of Excellence in Gambling*

*Director, Women and Addictions Core of Women’s Health Research*

*Director, Yale Impulsivity Research Program*

*Steven M. Southwick Professor of Psychiatry, Child Study, and Neuroscience*

**YALE UNIVERSITY**

*School of Medicine	One Church Street, Rm 726	New Haven, CT 06510*

*Email: marc.potenza@yale.edu*

May 5, 2025

Dear Sir or Madame,

Thank you for the request for additional information about our submission PONE-D-25-21070. Below are the points raised and responses.

1. Please complete all items on the Human Participants Research Checklist that are relevant for your submission, by following this link: https://nam12.safelinks.protection.outlook.com/?url=http%3A%2F%2Fjournals.plos.org%2Fplosone%2Fs%2Ffile%3Fid%3Ddc11%2FPLOSOne_Human_Subjects_Research_Checklist.docx&data=05%7C02%7Cmarc.potenza%40yale.edu%7Cf566c792590a423a31a408dd80fb9ee7%7Cdd8cbebb21394df8b4114e3e87abeb5c%7C0%7C0%7C638808540666535992%7CUnknown%7CTWFpbGZsb3d8eyJFbXB0eU1hcGkiOnRydWUsIlYiOiIwLjAuMDAwMCIsIlAiOiJXaW4zMiIsIkFOIjoiTWFpbCIsIldUIjoyfQ%3D%3D%7C0%7C%7C%7C&sdata=7xW5f2k3JKeCEp3PNelhE3SBdEYdHNuyZIdBcCLLqA4%3D&reserved=0 (Contact us at plosone@plos.org if you cannot access the document.) There may be overlap between the checklist items and other queries listed below; please address any duplicated queries both in your response email and on the checklist itself. Upload the completed Human Participants Research Checklist as file type “Other” when you re-submit your manuscript. This document is for internal journal use only and will not be published if your article is accepted. The requested information will help us to assess whether your submission complies with PLOS ONE’s policies and adheres to applicable reporting standards. Note that your manuscript may be rejected if you provide incomplete or inadequate responses to the checklist questions and that changing the

‘Section/Category’ of your article does not affect this requirement.

**Response:** We have completed the Human Participants Research Checklist.

2. We note that you have indicated that you received funding for your study. If you received peer reviewed external funding, your submission may be considered for expedited editorial assessment. To be considered for expedited review, please upload the Funding letter you received, and English translation, as an ‘Other’ file. Please also provide proof that the funder administered independent peer review of your protocol. Examples of evidence include: a link to a description of the funder’s peer-review process; a letter from the funding agency confirming the independent peer review process; a list of the names and credentials of the peer reviewers (if available).

Please note that we will not consider the following types of funding for expedited peer review: Funding from an author’s institution, or any other non-independent source; Funding from a commercial source; Funding that was not granted for this specific project (e.g. general PhD funding or fellowship).

If you are unable to provide proof of peer-reviewed external funding for your study, please note this in your cover letter to avoid delays in processing your manuscript. More information related to the relevant journal policies is available from: [https://nam12.safelinks.protection.outlook.com/?url=https%3A%2F%2Fjournals.plos.org%2Fplosone%2Fs%2Fsubmission-guidelines%23loc-study-protocols&data=05%7C02%7Cmarc.potenza%40yale.edu%7Cf566c792590a423a31a408dd80fb9ee7%7Cdd8cbebb21394df8b4114e3e87abeb5c%7C0%7C0%7C638808540666556811%7CUnknown%7CTWFpbGZsb3d8eyJFbXB0eU1hcGkiOnRydWUsIlYiOiIwLjAuMDAwMCIsIlAiOiJXaW4zMiIsIkFOIjoiTWFpbCIsIldUIjoyfQ%3D%3D%7C0%7C%7C%7C&sdata=xhtjvIIUjQKcMY%2BSTF%2FHnehfXQxDTwGQueyP4%2BV4USs%3D&reserved=0].

Financial Disclosure: R21DA058713

T32DA022975

K12DA00167

**Response:** We have uploaded the notice of award for the grant R21DA058713. Like other NIH grants, it has undergone rigorous peer-review. A description of the project can be found at NIH Reporter at: <https://reporter.nih.gov/search/FK_E-3bLu0SW76XVbI5Z7w/project-details/10887016>. We anticipate that the uploaded notice of award is for the use of the editors/reviewers and not to be included in the publication as most readers of PLoS One may find the relevant information online on the NIH Reporter website at the link above.

3. Please note that the Study Protocol article type is only suitable for proposals of studies that have not yet started or that are ongoing. Studies that have completed either recruitment or data collection will not be considered. For further information, please see https://nam12.safelinks.protection.outlook.com/?url=https%3A%2F%2Fjournals.plos.org%2Fplosone%2Fs%2Fsubmission-guidelines%23loc-study-protocols&data=05%7C02%7Cmarc.potenza%40yale.edu%7Cf566c792590a423a31a408dd80fb9ee7%7Cdd8cbebb21394df8b4114e3e87abeb5c%7C0%7C0%7C638808540666567346%7CUnknown%7CTWFpbGZsb3d8eyJFbXB0eU1hcGkiOnRydWUsIlYiOiIwLjAuMDAwMCIsIlAiOiJXaW4zMiIsIkFOIjoiTWFpbCIsIldUIjoyfQ%3D%3D%7C0%7C%7C%7C&sdata=mqlOIHizKd4uXXZq7eoqyf4uEa9124JBRuauYhG6J%2F8%3D&reserved=0. Please ensure that your Methods section reports the status and timeline of the study, including an estimate of when A) participant recruitment will be completed (if applicable), B) data collection will be completed and C) results are expected. If any of these stages have already been completed, this should be indicated in the Methods section.

**Response:** As of today, recruitment has not commenced. We anticipate completion of recruitment by April 2026, with results in the six months following.

4. We note you did not include a copy of the original, (signed or stamped) dated IRB/research ethics committee approval letter or waiver for all study sites as an ‘Other’ file. In keeping with our submission guidelines, please upload the approval letter or waiver as an ‘Other’ file. More information related to the relevant journal policies is available from: [https://nam12.safelinks.protection.outlook.com/?url=https%3A%2F%2Fjournals.plos.org%2Fplosone%2Fs%2Fsubmission-guidelines%23loc-guidelines-for-specific-study-types&data=05%7C02%7Cmarc.potenza%40yale.edu%7Cf566c792590a423a31a408dd80fb9ee7%7Cdd8cbebb21394df8b4114e3e87abeb5c%7C0%7C0%7C638808540666577317%7CUnknown%7CTWFpbGZsb3d8eyJFbXB0eU1hcGkiOnRydWUsIlYiOiIwLjAuMDAwMCIsIlAiOiJXaW4zMiIsIkFOIjoiTWFpbCIsIldUIjoyfQ%3D%3D%7C0%7C%7C%7C&sdata=U1QMzBv9TuKqRQzwJpHGBqoAS1YZDza9jh8sPmOKd%2F4%3D&reserved=0]

**Response:** We have now included a copy of the signed and dated approval letter, as described above.

5. Please upload a copy of your study protocol that was approved by your ethics committee/IRB as a Supporting Information file. By the study protocol, we mean the complete and detailed plan for the conduct and analysis of the trial approved by the ethics committee/IRB. Please send this in the original language. If this is in a language other than English, please also provide a translation. [https://nam12.safelinks.protection.outlook.com/?url=https%3A%2F%2Fjournals.plos.org%2Fplosone%2Fs%2Fsubmission-guidelines%23loc-guidelines-for-specific-study-types&data=05%7C02%7Cmarc.potenza%40yale.edu%7Cf566c792590a423a31a408dd80fb9ee7%7Cdd8cbebb21394df8b4114e3e87abeb5c%7C0%7C0%7C638808540666587515%7CUnknown%7CTWFpbGZsb3d8eyJFbXB0eU1hcGkiOnRydWUsIlYiOiIwLjAuMDAwMCIsIlAiOiJXaW4zMiIsIkFOIjoiTWFpbCIsIldUIjoyfQ%3D%3D%7C0%7C%7C%7C&sdata=X6a0CxUxfMjoydMEcaEYl3%2BIYUo2RfGOxfqH3o0Ka%2Bw%3D&reserved=0]

**Response:** We have now included a copy of the IRB-approved study protocol, as described above.

Sincerely,

Marc N. Potenza, MD, PhD

Steven M. Southwick Professor of Psychiatry in the Child Study Center and of Neuroscience

Yale School of Medicine
